# Supplementary figures and images for: Predicting antifolate resistance in the unculturable fungal pathogen Pneumocystis jirovecii
Source: PLoS Genet. 2026 May 27;22(5):e1012163. doi: 10.1371/journal.pgen.1012163 (PMC13241008; doi:10.1371/journal.pgen.1012163)

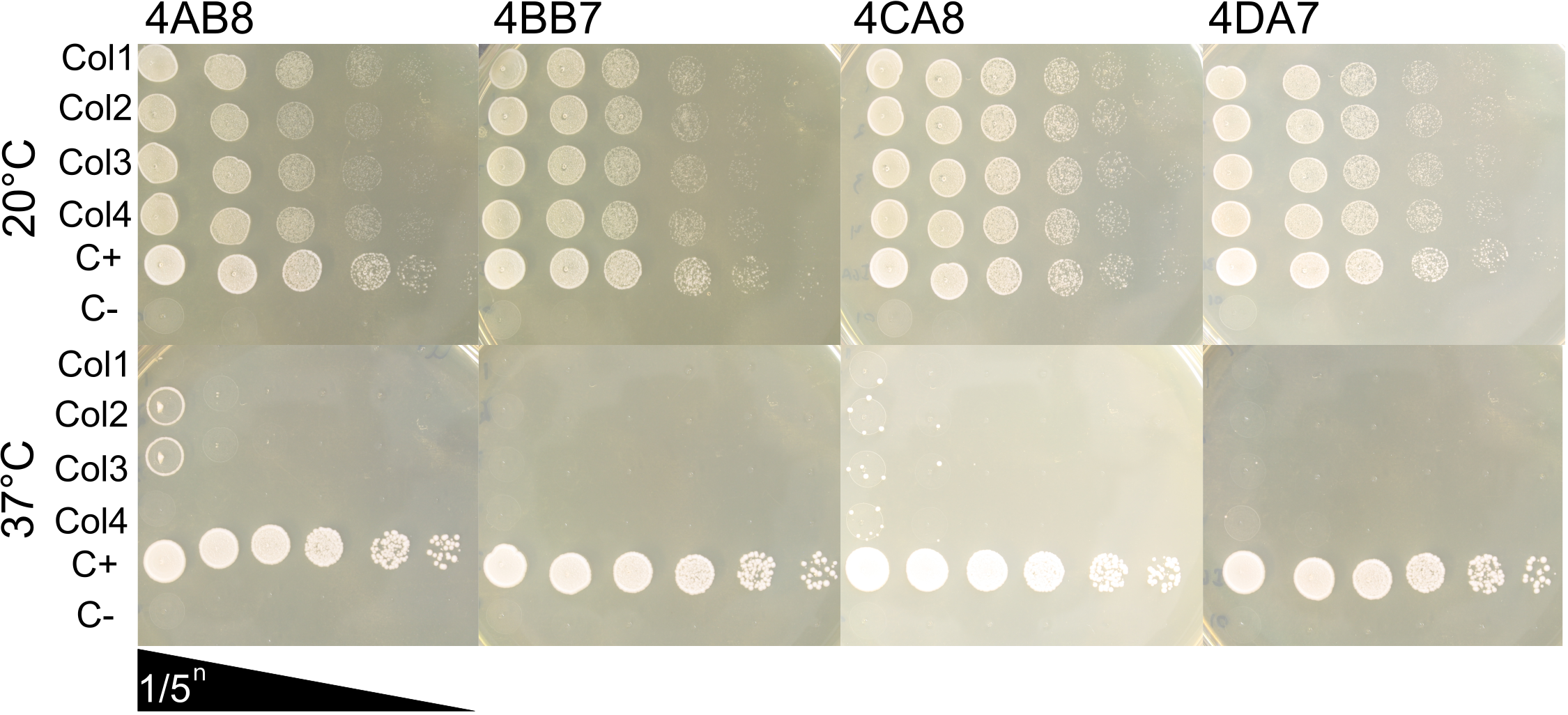

Supplement: S1 Fig — From each TS-DHFR strain from the temperature sensitive allele collection [64], samples from the collection glycerol stock were spread on YPD + G418 plates. From each biological replicate, four isolated colonies were selected and grown overnight in YPD + G418 liquid media. Cultures were then diluted to 1.0 OD600 and spotted on YPD + G418 plates with 5-fold serial dilutions. One plate was incubated at 20°C (permissive condition, top) and at 37°C (selection condition, bottom) for 72h. On each plate, strain IGA130 (DHFR-WT) and strain FDR0001 (Δdhfr, inducible DfrB1 to allow for growth) were used as positive and negative growth controls, respectively [36]. (TIFF) [file pgen.1012163.s014.tiff]

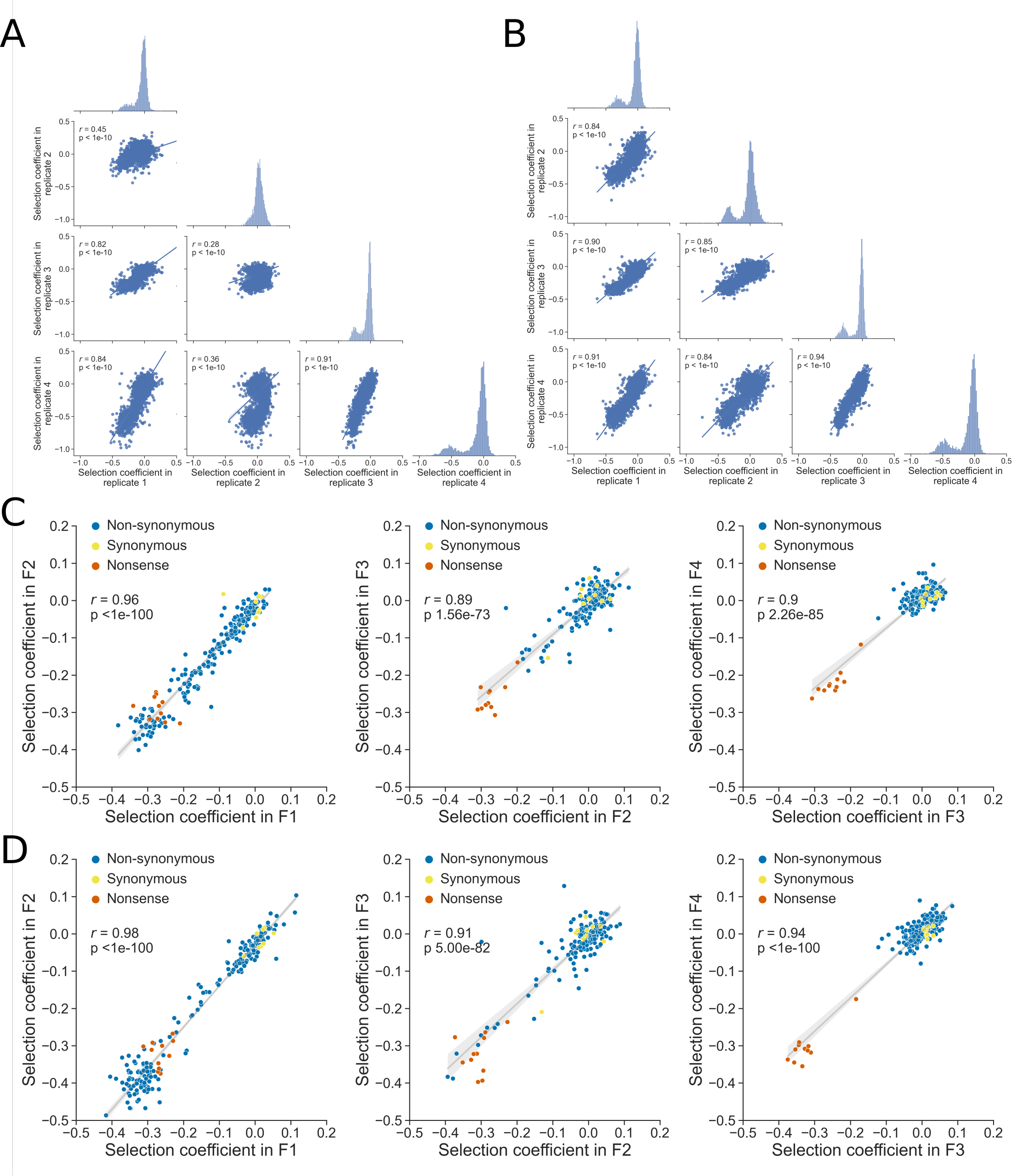

Supplement: S2 Fig — A) Scatterplots of selection coefficients for mutated codons in the different biological replicates at 20°C. Axes are scaled to allow comparison of the different conditions. Density plots show the distribution of selection coefficients for the replicate on the x-axis. B) Scatterplots of selection coefficients for mutated codons in the different biological replicates at 37°C. Axes are scaled to allow comparison of the different conditions. C) Scatterplots of selection coefficients for amino acids present in the overlap between the different fragments at 20°C to ensure good correlation between the different fragments. D) Scatterplots of selection coefficients for amino acids present in the overlap between the different fragments at 37°C to ensure good correlation between the different fragments. Scatterplots for each fragment overlap show similar patterns at 20°C and at 37°C. (TIFF) [file pgen.1012163.s015.tiff]

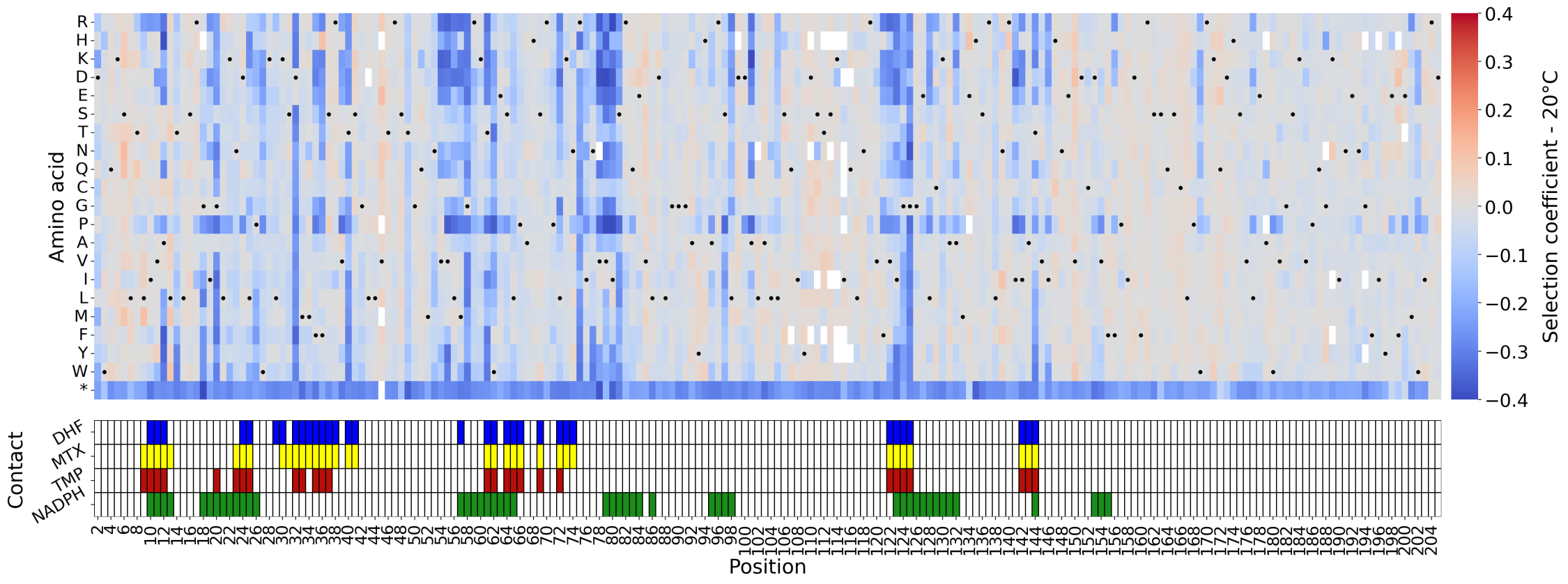

Supplement: S3 Fig — Selection coefficient is measured relative to the median of silent mutations. Positions of contacting residues along PjDHFR (PDB: 3 CD2 (MTX and NADPH), 4 CD2 (DHF) and 1DYR (TMP)). Contact was established as amino acids with an α-carbon located less than 8 Å from MTX, DHF, TMP or NADPH. Black dots represent the wild-type sequence. (TIFF) [file pgen.1012163.s016.tiff]

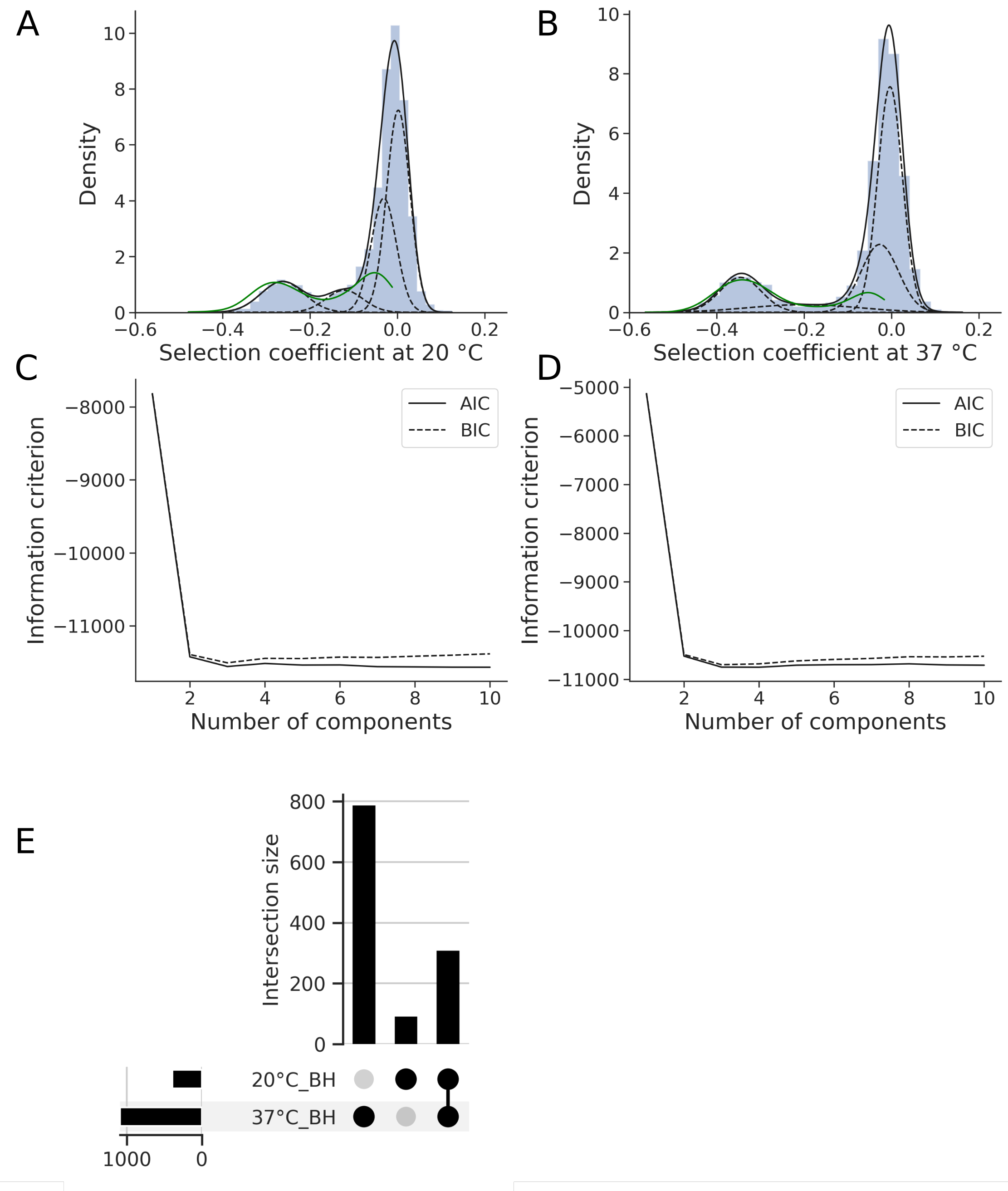

Supplement: S4 Fig — Optimization of information criteria for A) 20°C and B) 37°C. Best Gaussian mixture model (dashed lines represent underlying Gaussians) to recapitulate the underlying distribution (black line/ histogram) of C) 20°C and D) 37°C. Density curve of significant mutants for Benjamini-Hochberg (green) correction is visible. E) Intersection between statistically significant mutants in 20°C and 37°C. Upset plot showing the intersections of the Benjamini Hochberg-FDR corrected groups (control for the false discovery rate of significantly resistant mutants at 95% confidence). (TIFF) [file pgen.1012163.s017.tiff]

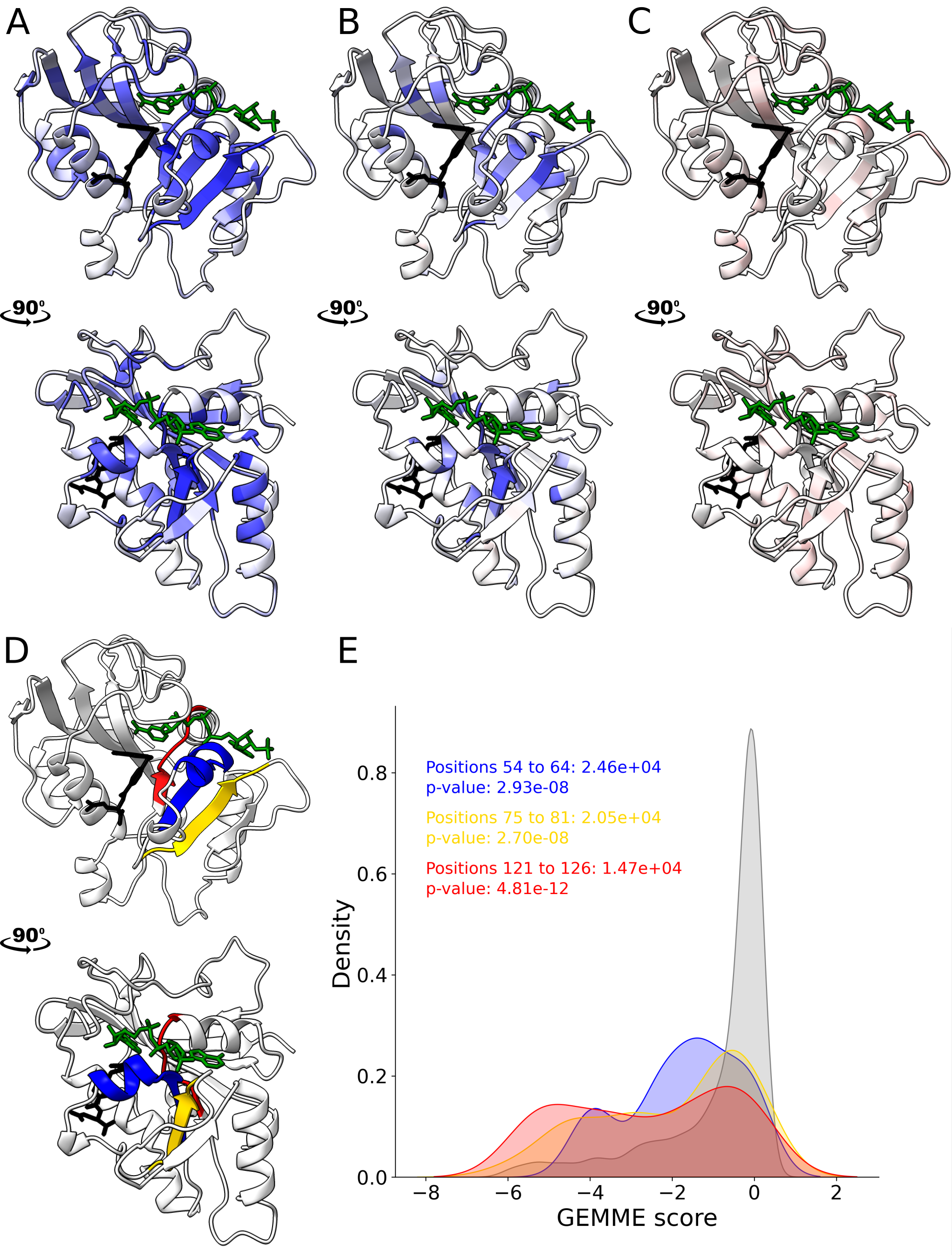

Supplement: S5 Fig — A) Position-wise minimum score, colored as in Fig 2A. B) Position-wise median score. C) Position-wise maximum score. D) Visualization of the region with a high rate of SDE. Positions 54–64 are colored in blue, positions 75–81 in yellow and 121–126 in red. E) Distribution of GEMMECombined scores for all positions in grey, positions 54–64 are colored in blue, positions 75–81 in yellow and 121–126 in red. One-sided Mann-Whitney U scores and p-values for each distribution are shown. Mutations in these regions are predicted as being more likely to be deleterious than most mutations across the protein. NADPH is colored in green, and folate is colored in black. (TIFF) [file pgen.1012163.s018.tiff]

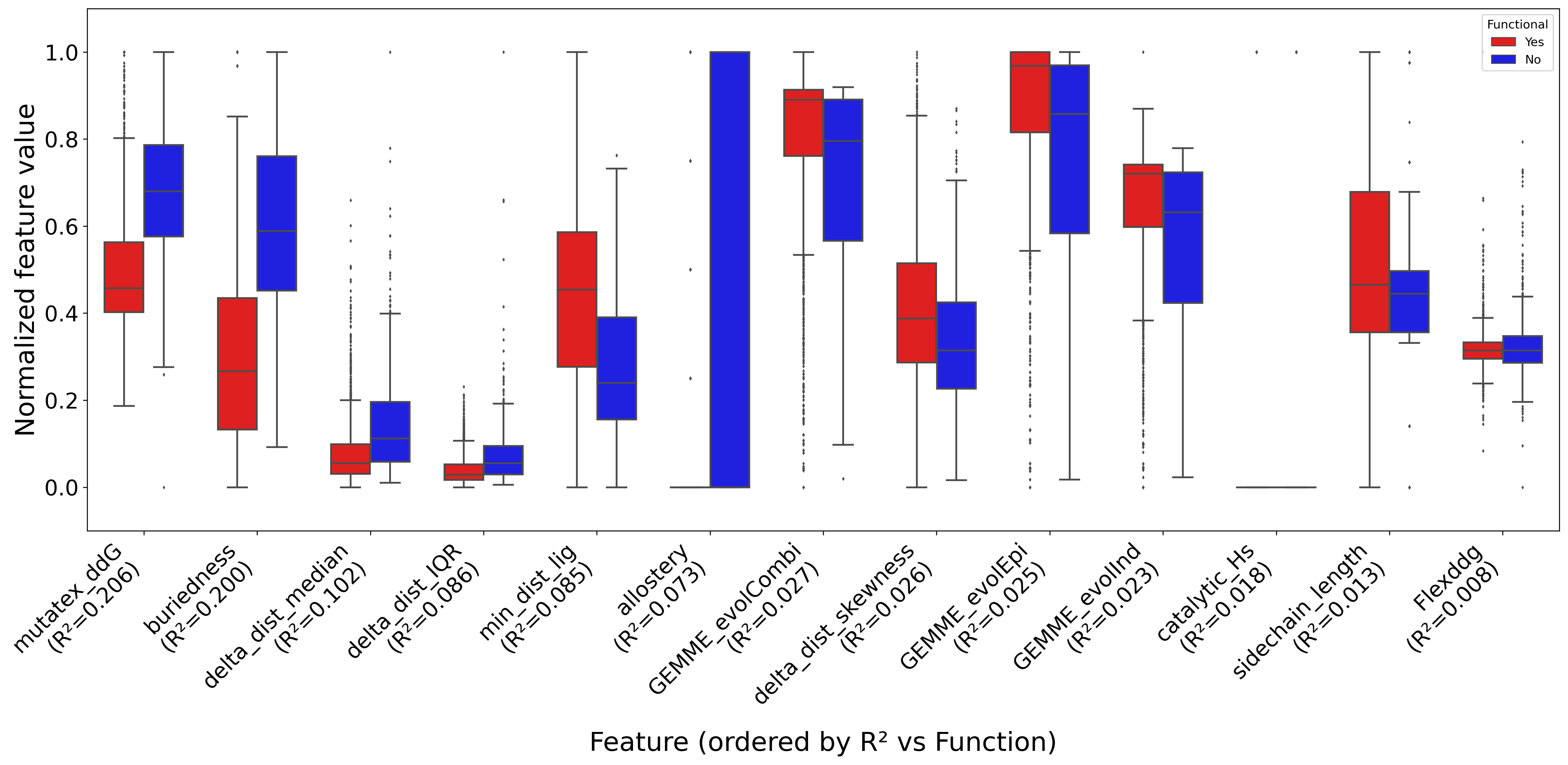

Supplement: S6 Fig — Distribution of feature values are normalized between 0 and 1 for each feature used in the analysis, separated by variants classified as functional or non-functional in the DMS assay. Features are ordered according to the proportion of variance in functional scores explained by each feature individually (R²), calculated using linear regression against the experimentally measured functional scores. This highlights the relative explanatory power of the computational features. For this analysis, the FlexddG data used was computed using MTX as a ligand. (TIFF) [file pgen.1012163.s019.tiff]

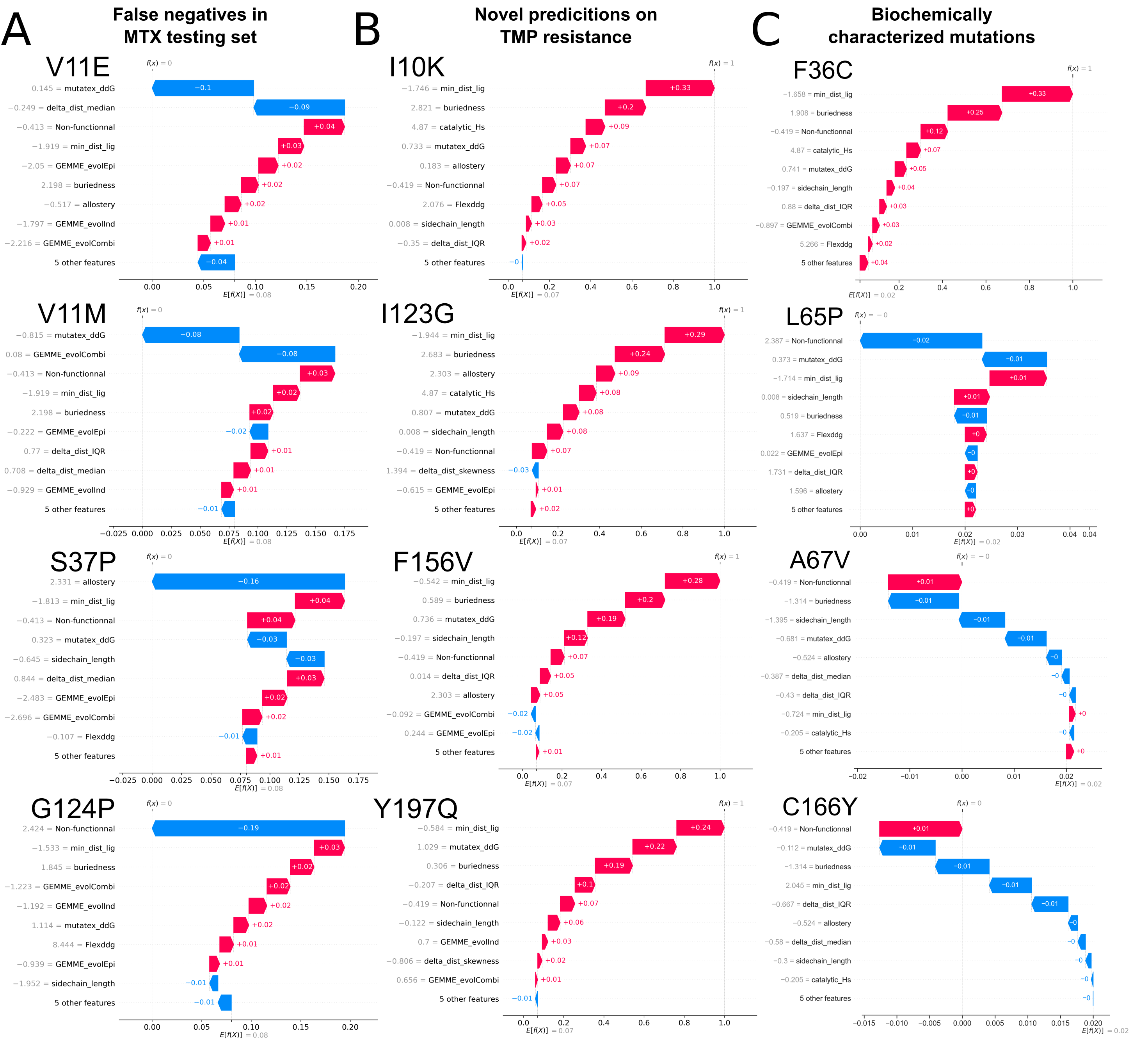

Supplement: S7 Fig — A) Waterfall plots detailing classification decisions for mutants that were classified as false negatives in the testing dataset for MTX resistance (leading to resistance experimentally but not being predicted as leading to resistance by the model.) V11E (Prediction probability of leading to MTX resistance of 0.33) and V11M (0.36) are both predicted as being destabilizing by MutateX, S37P (0.36) misclassification is mainly driven by its classification as a high confidence allosteric mutation by [44], and G124P (0.25) was classified as being non-functional. B) Waterfall plots detailing classification decisions for mutants that were predicted by the models as leading to TMP resistance. I10K (probability of 0.94), F121H (0.52), I123G (0.82), and Y197Q (0.81) are all novel predictions made by the model (Not measured/predicted as leading to MTX resistance but predicted as leading to TMP resistance.) C) Waterfall plots detailing classification decisions for mutants that were observed either in this or in previous study and have been linked to TMP resistance in PjDHFR. F36C (Prediction probability of leading to TMP resistance of 0.91) was predicted by the model as leading to TMP resistance (and not observed as leading to MTX resistance), but not L65P (0.33), A67V (0.03) or C166Y (0.01). L65P was experimentally identified as being non-functional. A67V was classified as not leading to TMP resistance mainly because of its buriedness level, as well as effect on protein stability. C166Y was classified as not leading to TMP resistance because of its distance to the ligand, buriedness level and effect on protein stability. All SHAP values are calculated on z-score scaled data. (TIFF) [file pgen.1012163.s020.tiff]

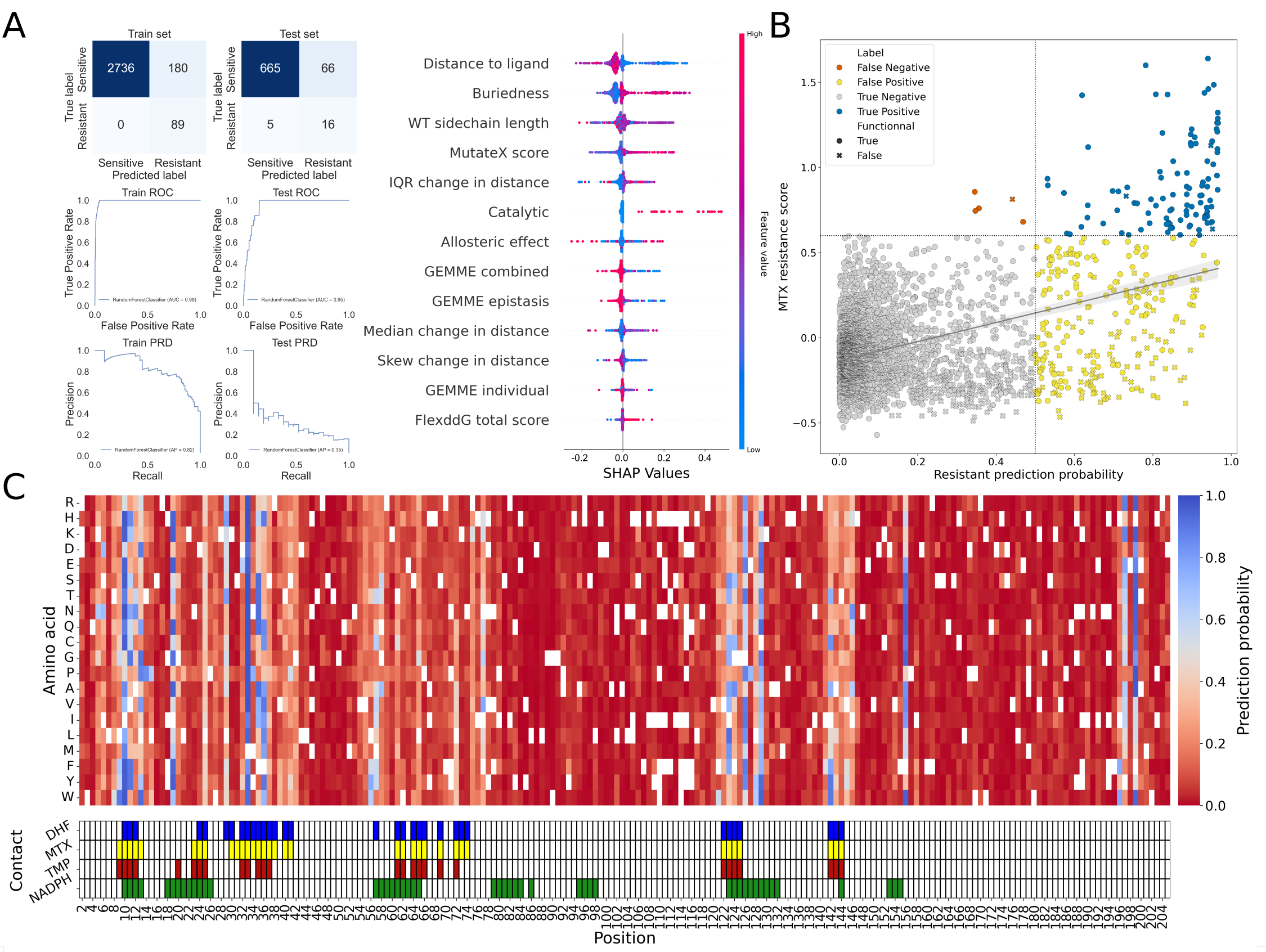

Supplement: S8 Fig — A) Confusion matrices and ROC/PRD curves for train and testing set splits, with SHAP value explainer plot, with relative impact of features on model decision. True positives and false negatives stayed mostly consistent with the complete model, but much more false positives were predicted, highlighting the importance of functional information. B) Scatterplot comparing ground truth from MTX DMS screening vs model’s predicted probability. True negatives in grey, true positives in blue, false positives in yellow and false negatives in orange. Mutations that were identified as functional in the function DMS screening have round markers and mutations identified as non-functional have X-shaped markers. X-shaped markers are much more numerous in the false positive quadrant. C) Heatmap with prediction probability of being resistant to MTX. Mutations with probability >0.5 are identified as resistance conferring, and mutations ≤ 0.5 are classified as sensitive. (TIFF) [file pgen.1012163.s021.tiff]

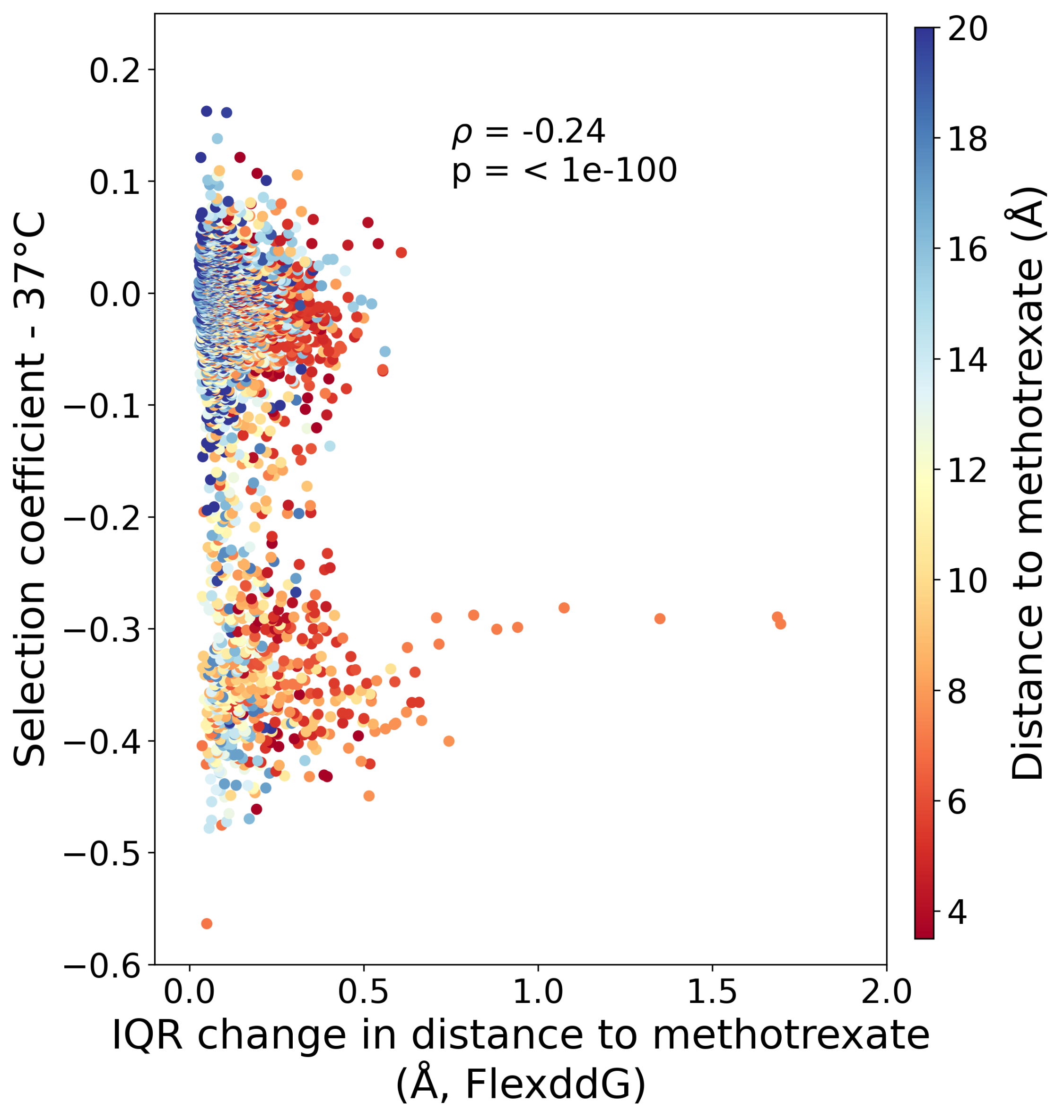

Supplement: S9 Fig — For each structure generated for all mutations during FlexddG saturation mutagenesis, the median position of the α-carbon of the wild-type structures was used as the baseline. This was compared to the position of the corresponding α-carbon of the mutated residue of all 100 structures for each mutant, resulting in a distribution of changes in distance between the wild-type α-carbon and the corresponding mutant’s α-carbon. From this distribution, we extracted the interquartile range of the distribution, representing the “flexibility” of the protein region caused by the mutation. A wide distribution (high IQR) means the mutated residue increases region flexibility, as the mutated region can occupy a broader range of positions further from its wild-type position. (TIFF) [file pgen.1012163.s022.tiff]
