# Supplementary material for: Predicting antifolate resistance in the unculturable fungal pathogen Pneumocystis jirovecii
Source: PLoS Genet. 2026 May 27;22(5):e1012163. doi: 10.1371/journal.pgen.1012163 (PMC13241008; doi:10.1371/journal.pgen.1012163)
Supplement: Attachment — Submitted filename: ResponseToReviews_18032026.docx [file pgen.1012163.s026.docx]

**Reviewers' comments:**

Reviewer's Responses to Questions

**Comments to the Authors:
Please note here if the review is uploaded as an attachment.**

Reviewer #1: SUMMARY
This study presents a framework for predicting antifolate resistance in Pneumocystis jirovecii, an unculturable fungal pathogen of major clinical consequence. By integrating a large deep mutational scanning data set, biophysical modeling, and machine-learning approaches, the authors train a predictive model for methotrexate resistance and extend it to predict trimethoprim resistance, which cannot be experimentally assayed in vivo. The work is technically strong, conceptually well motivated, and addresses a genuine gap in our ability to study resistance evolution in hard-to-study pathogens.

More importantly, this study presents a responsible use of machine learning methodology in a world where I see too many papers that utilize machine learning in a careless manner. This could serve as a standard for how we use these approaches moving forward. That said, there are some significant challenges with the study in its current form.
My major concerns with this study are that it is unfocused. It reads largely as a technological expedition, which there is nothing wrong with in the pure sense. However, if this is a methods paper, then it should be written as such. And a methods paper often requires that a method be tested in different circumstances, across different data sets.

Alternatively, if this is a paper about genetics or evolution, then it should be written as such. I recognize that the inability to validate the predictions are an impediment to this paper’s biological conclusions. And I don’t want to be the sort of reviewer that makes this sort of demand (which would be a tremendous amount of work), but something must be done to make this paper much more than an interesting machine learning exercise that may have some biological implications. As it stands, the study does a very poor job of teaching me anything in particular about the natural world.

For example, the conclusion reads as if the paper is an engineering proof-of-concept. What, specifically, about the biology of drug resistance, did we learn? Is the point that mutation effects in a different drug can be predicted using a large-scale DMS data set, and machine learning algorithms are used? What does this say about biology and genetics other than that machine learning allows us to predict things? I think this study is successful in using an original approach (the biophysical components, for example, are great). But there is no narrative and the paper isn’t organized in a manner that allows the reader to follow any particular theme.

**##GENERAL RESPONSE:** We thank the reviewer for highlighting concerns regarding the focus, narrative, and biological implications of the original manuscript. In response, we have revised the introduction and the conclusion to frame the study more clearly around a central biological question, being how antifolate resistance evolves in *Pj*DHFR under functional constraints, and to what extent resistance determinants can be generalized across similar antifolates targeting the same enzyme.

In the revised introduction, we emphasize that this work is not intended primarily as a methodological or machine-learning exercise, but as an investigation of the biological constraints governing resistance evolution in an unculturable pathogen. We clarify that resistance-conferring mutations must preserve a level of DHFR function, explicitly introducing this trade-off as a guiding biological principle of the study. To address concerns about generalization we reframe how results from methotrexate can be transferred to trimethoprim as a test of cross-resistance, highlighting that while MTX and TMP share a binding site and mechanism, the extent to which resistance is generalizable remains an open question that depends on molecular, functional, and systems constraints. Within this framework, the machine-learning approach is explicitly stated as a tool to help understand these biological questions rather than the goal.

We also revised the conclusion to explicitly state the biological insights and conclusions derived from the results. The revised conclusion highlights that the main contribution of our work lies in identifying the biological constraints that govern antifolate resistance, particularly the requirement that resistance-conferring mutations preserve function. We further clarify that the machine-learning framework serves as an integrative approach to reveal functional and mechanistic non-linear trade-offs, rather than only as a predictive model. We also explicitly discuss what these findings imply for the predictability and limits of resistance evolution and for the generalizability of resistance mechanisms across related antifolate drugs, to focus on what is learned about the natural world rather than focusing primarily on machine learning outcomes.

See the main modifications added to the main text in regard to this comment:

The authors do discuss some specific mutations and their character. The biophysical features are interesting. But the results and discussion sections overall read as sporadic takes on various findings, mostly focusing on the ability of ML to predict mutations. What does this say for predictive evolution of resistance? Or for drug design? Or for how we think about tradeoffs in protein evolution? The study need not focus on one thing, but it currently reads as if a large technological feat was pulled off (impressive) and the authors then searched for a relevant question, after the results came in. Again, this is not a bad thing, but as written, the study doesn’t read with the sorts of cohesion that it could.
I suggest the authors write the manuscript from the perspective of the challenges in identifying cross-resistance. And/or the relevance of the findings for medicinal chemistry, or fields interested in identifying how evolution affects resistance mechanisms across drugs. I think this can be achieved with different foci.

**## Response:** We thank the reviewer for their comments. We agree that the study focused on the output from the ML model rather than interpretation and biological insights. However, we believe that it falls within the scope topics of PLoS Genetics: Computational, statistical and AI-driven genomics and Microbial, plant and host-microbe genomics. Nevertheless, we have reframed the manuscript, especially the introduction and discussion, to focus further on the biological insights gleaned from feature analysis from the model, functional tradeoffs, the importance of the experimental DMS data for model performance as well as the limits of cross-resistance and transferability from one drug to another. Thus, we believe that we have emphasized the biological relevance and conclusions of this study, as requested. ##

See the main modifications added to the Discussion section in regard to this comment. Modifications from the previous comment in the Introduction and Conclusions sections are also relevant here:

Relatedly, I think the study would benefit tremendously from a table that orients the reader to their choice of ML protocol. Too often I see these papers where the method is used and it is not clear to the reader why a given algorithm was used. Here is what I suggest:

A table or box that explains what random forest does, why it is appropriate for the data structure, and substantive scholarly for this. In addition, the authors should highlight other ML methods that have been used for other related studies, and why they do not capture what is necessary for the specific questions posted.
Moreover, the study should have a different summarizing table that highlights the specific biological points, and implications. This would, of course, be tied to rewrites that better organize the study into its methodological and biological implications.

**## Response:** We thank the reviewer for this comment. While we agree that such a table could help readers orient themselves within the manuscript, the aim of this paper is not to provide introductory information based on machine learning or the different types of such. We have, however, clarified why we have used a Random Forest model over other methods, such as black-boxes deep learning. We hope that this revision satisfies the reviewer. ##

OTHER POINTS:
The abstract is very long and confusing. I’d almost recommend a clinical-style abstract where the authors delineate exactly what the question is, what methods they used, the most notable results, and implications. I actually think this should be standard practice in all machine-learning style papers, as it clearly delineates the data science/computational aspects from the true basic science essence. But this is especially true for this study, where the abstract is difficult to follow.

**## Response:** We thank the reviewer for this insightful suggestion. We have revised the abstract in a clinical style and believe that it does indeed help clarify and better set expectations for the content of the paper. ##

VERY MINOR COMMENTS:
Line 105: “The DHFR” , Remove “The” (I think)
Abstract: 100-folds should be 100-fold
LINE 175: I could be wrong, but “temperature sensitive (TS) DHFR constructions” should be “temperature sensitive (TS) DHFR constructs.”
Line 237: validating it’s a high degree of importance' should be 'validating its high degree of importance.
Figure 1 caption: PBD: 3CD2 should be PDB: 3CD2
Line 383: selection coefficient a 37 °C should be 'selection coefficient at 37 °C.
Line 595: LOFs mutations is wrong, I think

**## Response:** We thank the reviewer for pointing out the complexity of the abstract. It has been revised in a clinical style to highlight the different aspects of the study and the key points more clearly from each. We also corrected all other minor comments. ##

Reviewer #2: The manuscript by Rouleau et al. uses deep mutational scanning plus structural modeling and machine learning to predict which mutations in Pneumocystis jirovecii dihydrofolate reductase (PjDHFR) can confer resistance to antifolate drugs, especially trimethoprim (TMP), in a pathogen that cannot be cultured in vitro. Because P. jirovecii cannot be grown in the lab, the authors express its DHFR in yeast and perform deep mutational scanning to measure how all single amino‑acid changes affect enzyme function and resistance to methotrexate (MTX), a related antifolate. The work nicely combines experimental DMS for function and MTX resistance, multiple structure‑based predictors (MutateX/FoldX, FlexddG, GEMME), and machine learning into a coherent pipeline tailored to an unculturable pathogen. The analysis identifies distance to ligand, functional status, buriedness, stability, and flexibility as key features, which are aligned with biochemical intuition.

Overall, I found the work to be technically strong, clearly written, which addresses a clinically relevant problem with a workaround to experimental intractability. The integration of functional DMS data as a key feature is a major strength and differentiates this work from prior purely in silico approaches. However, several conceptual and interpretational issues should be addressed to strengthen the manuscript and align claims more tightly with the evidence presented. I will list a few concerns which I hope to help authors strengthen this interesting work.

Major comments/suggestions:

**## Response**: We thank the reviewer for this comment. We have reframed much of the focus of the paper to emphasize the aspects raised by the reviewer’s comment. Several sections have been added to the introduction, results and discussion sections to clarify and focus the conclusions from the study. Modifications have been included to address all four major points raised by the reviewer. We believe that the modifications emphasize the relevant points, reframe the main take home messages from the manuscript, and highlight design choices and limitations inherent to the model. We hope that the modifications are satisfactory.

1) While the manuscript emphasizes predictive performance, the most compelling contribution is arguably mechanistic insight into resistance determinants, not prediction alone. Otherwise, one would expect authors to have checked several ML methods including deep neural networks. The random forest model is repeatedly justified by accuracy metrics (balanced accuracy, ROC, PRD), but the biological interpretation of feature importance (distance to ligand, flexibility, functionality) is where the work truly advances the field. I recommend reframing parts of the Results and Discussion to emphasize that the ML model is primarily a tool for integrating heterogeneous and non-linear mechanistic features, rather than a black-box predictor.

\## Modifications in response to reviewer 2, comment 1:

2) The central assumption that MTX resistance mechanisms generalize to TMP resistance because both are antifolates is plausible. Although structural similarity and shared binding sites are discussed, the manuscript would benefit from a clearer articulation of where this analogy might break down. In light of the Popperian falsifiability, is there a reason to believe that such transferability might not work?

\## Modifications in response to reviewer 2, comment 2:

3) The authors correctly discuss class imbalance. However, the decision threshold of 0.5 for resistance classification is somewhat arbitrary given the severe imbalance (≈3% resistant). Would it make sense to consider stratifying predictions into high-, medium-, and low-confidence classes and explicitly be cautious against over-interpreting marginal predictions?

\## Modifications in response to reviewer 2, comment 3:

4) One of the most important findings is that DMS data dramatically improves resistance prediction. This point has implications far beyond P. jirovecii and deserves stronger emphasis. I might have overlooked but the manuscript could more clearly state that purely computational resistance predictors are fundamentally limited without functional data.

\## Modifications in response to reviewer 2, comment 3:

Specific comments in relation to the manuscript’s content:

5) The authors state:
“We define these amino acid substitutions as loss-of-function (LOF) mutations.”

This terminology is potentially too strong and may be misleading. In the broader genetics literature, loss-of-function typically refers to classical alleles—such as truncating, nonsense, or frameshift variants—that abolish gene function. In contrast, the variants described here are missense substitutions whose classification as LOF is based on reduced fitness in a specific functional complementation assay in S. cerevisiae under defined temperature and expression conditions. As such, these mutations are more accurately described as strongly deleterious in this assay, or functionally impaired, rather than bona fide LOF alleles.

\## **Response**: We agree that the terminology used was strong, and could be misleading in the light of classical genetics. We have modified LOF and replaced it with strongly deleterious effects (SDE) in the text.##

6) The figure legends are currently insufficient to fully explain the information presented in the figures. Ideally, each figure should be interpretable on its own, solely by reading the legend; however, in several cases this is not possible. For instance, in Figure 2, the track immediately below the heatmap appears to encode contacts between PjDHFR and different ligands, using multiple colors, but this is neither clearly explained nor justified in the legend. Moreover, the same or similar colors are later reused to represent different categories of amino acid substitutions, which may lead to confusion. I recommend that the authors carefully revise all figure legends to ensure they comprehensively describe each visual element and use consistent, unambiguous color schemes, so that the figures are easy to interpret at a glance.

\## **Response**: We thank the reviewer for pointing out that the figure legends did not allow for a full understanding of the figure’s contents. We have modified the legends of figures 2-3-4 to be clearer and allow for a full interpretation of the figures. ##

7) The opening of the Results section revisits concepts that are fundamental and already well established in the literature. For example, the observation that highly conserved residues are more likely to yield deleterious substitutions is a long-standing principle in functional variant interpretation, dating back at least to methods such as SIFT. While I am not suggesting that this section be shortened, the authors may reconsider the emphasis and highlight aspects of their results that are more conceptually interesting or novel. One particularly compelling point is the explicit requirement that resistance-conferring mutations must preserve a minimal level of protein function. As the authors note, even mutations that strongly disrupt inhibitor binding will not confer resistance if they simultaneously abolish essential enzymatic activity, as such variants cannot support cell viability. This functional–resistance constraint is less explicitly discussed in much of the existing literature and could be more strongly emphasized as an important conceptual insight emerging from their dataset.

**## Response:** We thank the reviewer for this comment. We agree that revisiting this concept is redundant at large. However, we believe that it is the first such analysis done for a fungal DHFR, and have therefore decided to not reduce this section too much. However, we have also added statements that reframe it within the scope of the paper, especially regarding the importance of identifying such regions and mutational effects on model performance and predictive power, as well as its importance within the field of antifungal resistance. We hope that this reframing satisfies the reviewer.

See the main modifications added to the main text in regards to this comment:

8) The rationale starting around line 298 provides a thorough and well-reasoned rationale for why no single modeling approach can fully capture protein dynamics and function, and why different methods are expected to explain complementary aspects of the DMS results. However, this framing naturally raises the question of the most appropriate statistical framework to quantify these contributions. In the current version, this is supported by correlations, which, while informative, offer a limited view of explanatory power. It may therefore be valuable to complement these analyses with more quantitative approaches, such as estimating the proportion of variance in functional scores explained by each modeling method (e.g., via regression or variance partitioning), or performing enrichment analyses to test whether mutants with high or low functional scores are overrepresented among variants predicted as deleterious or destabilizing by each method. Simple contingency-table–based tests (e.g., Fisher’s exact test) could provide an intuitive and statistically grounded way to assess the overlap between experimental outcomes and model predictions, and would strengthen the link between the conceptual argument and the quantitative evidence.

**## Response:** We thank the reviewer for this comment regarding the need for a more quantitative framework to assess the contribution of different modeling approaches. We have expanded this section to complement correlation analyses with regression-based quantification of explanatory power.

Specifically, we now estimate the proportion of variance in experimentally measured functional scores explained by each computational feature using linear regression (R²). These results are presented in a new supplementary figure (Fig. S6). We show that predicted changes in folding free energy (MutateX) explain the largest fraction of variance, followed by residue buriedness and flexibility-related features. This analysis supports our previous observation that protein stability is the strongest predictor of function, while also demonstrating that no individual feature captures full mutational effects. This provides a more explicit statistical framework supporting the conclusion that different modeling approaches capture complementary aspects of protein function. This also strengthens the link between better computational predictions and experimental measurements of function.

See the main modifications added to the main text in regards to this comment:

9) In the section that starts on the random forest model (lines 334 onwards), authors write:
“From various experiments and computational analyses, we believed that we had gathered comprehensive data on different aspects of protein function, resistance, and mechanisms.“
However, the previous section ultimately concludes with the observation that protein stability appears to be the strongest single predictor of DMS-derived functional scores. A more effective way to introduce the subsequent section might be to frame the use of the random forest model as a means of capturing non-linear interactions among multiple features, rather than as a tool primarily aimed at maximizing predictive performance. More generally, while the predictive accuracy of machine-learning models is clearly valuable, their greatest strength in this context may lie in their potential to illuminate underlying biological mechanisms and functional constraints, which I also raised as a major comment. Emphasizing this interpretability-driven perspective, rather than focusing predominantly on predictive power, could strengthen the conceptual framing of this section—though this may ultimately be a matter of emphasis and preference. In this light, it would also be helpful for the authors to explicitly justify their choice of a relatively interpretable machine-learning method such as random forest, as opposed to more opaque but potentially more predictive approaches (e.g., deep neural networks).

**## Response:** We thank the reviewer for this comment. We have revised the opening of the random forest section to better frame the model as a tool to capture non-linear interactions among complementary features, rather than primarily to maximize predictive performance. While protein stability did emerge as the strongest single predictor, we now emphasize that no individual feature is sufficient to explain resistance, further supporting the integrating approach around which we now focus the paper.

We also clarify that the goal of the model is to provide biological insight into the constraints shaping resistance and not just prediction. In this context, we explicitly justify the use of a random forest model as a balance between predictive power and interpretability, allowing us to extract feature importance, in contrast to black box methods.

See the main modifications added to the main text in regards to this comment:

10) In several places, the term “resistance” is used interchangeably for biochemical affinity changes and organism-level resistance. Would it make sense to consider clearer distinctions? Also in places, it is written that “mutations are identified as resistant” which I would change to "resistance conferring”.

**## Response:** We agree with the reviewer that the terms were used interchangeably, which led to confusing sections. We have modified the main text in several places to clarify the differences between affinity changes and organism resistance to antifolates. We hope that the modifications throughout the full manuscript clarify manuscript reading.

11) Figure 4 is conceptually very important but is not presented well and gives the impression that it is made in a hurry. It would be nice to show a few residues, compare such residues with the MTX resistance pattern and also map them to the protein structure.

**## Response:** We thank the reviewer for this comment, and wholeheartedly agree. We struggled a fair amount in regards to how to present the results in figure 4, and finally settled for a heatmap, as it is the most common way to present DMS results, and was in line with the style of the manuscript. However, we agree that it limits the biological interpretation of one of the main results from this manuscript. We have completely overhauled figure 4, with focus on PjDHFR structure and how the predicted scores and thresholds map across the structure.

See the main modifications added to the main text and figure 4:
